# Multisensory Gains in Simple Detection Predict Global Cognition in Schoolchildren

**DOI:** 10.1038/s41598-020-58329-4

**Published:** 2020-02-04

**Authors:** Solange Denervaud, Edouard Gentaz, Pawel J. Matusz, Micah M. Murray

**Affiliations:** 1 The Laboratory for Investigative Neurophysiology (The LINE), Department of Radiology, Vaudois University Hospital Center and University of Lausanne, Lausanne, Switzerland; 2 The Center for Affective Sciences (CISA), University of Geneva, Geneva, Switzerland; 3 Faculty of Psychology and Educational Sciences (FAPSE), University of Geneva, Geneva, Switzerland; 4 Information Systems Institute at the University of Applied Sciences Western Switzerland (HES-SO Valais), 3960 Sierre, Switzerland; 5 Department of Hearing and Speech Sciences, Vanderbilt University Medical Center, Nashville, TN USA; 6 Department of Ophthalmology, Fondation Asile des aveugles and University of Lausanne, Lausanne, Switzerland; 7 Sensory, Cognitive and Perceptual Neuroscience Section, Center for Biomedical Imaging (CIBM) of Lausanne and Geneva, Lausanne, Switzerland

**Keywords:** Perception, Human behaviour

## Abstract

The capacity to integrate information from different senses is central for coherent perception across the lifespan from infancy onwards. Later in life, multisensory processes are related to cognitive functions, such as speech or social communication. During learning, multisensory processes can in fact enhance subsequent recognition memory for unisensory objects. These benefits can even be predicted; adults’ recognition memory performance is shaped by earlier responses in the same task to multisensory – but not unisensory – information. Everyday environments where learning occurs, such as classrooms, are inherently multisensory in nature. Multisensory processes may therefore scaffold healthy cognitive development. Here, we provide the first evidence of a predictive relationship between multisensory benefits in simple detection and higher-level cognition that is present already in schoolchildren. Multiple regression analyses indicated that the extent to which a child (N = 68; aged 4.5–15years) exhibited multisensory benefits on a simple detection task not only predicted benefits on a continuous recognition task involving naturalistic objects (*p* = 0.009), even when controlling for age, but also the same relative multisensory benefit also predicted working memory scores (*p* = 0.023) and fluid intelligence scores (*p* = 0.033) as measured using age-standardised test batteries. By contrast, gains in unisensory detection did not show significant prediction of any of the above global cognition measures. Our findings show that low-level multisensory processes predict higher-order memory and cognition already during childhood, even if still subject to ongoing maturation. These results call for revision of traditional models of cognitive development (and likely also education) to account for the role of multisensory processing, while also opening exciting opportunities to facilitate early learning through multisensory programs. More generally, these data suggest that a simple detection task could provide direct insights into the integrity of global cognition in schoolchildren and could be further developed as a readily-implemented and cost-effective screening tool for neurodevelopmental disorders, particularly in cases when standard neuropsychological tests are infeasible or unavailable.

## Introduction

When a child wishes to cross the street, simply looking left and right for incoming cars is not always sufficient to make a safe choice. Sensitivity to additional cues, like the noise generated by an approaching car, will also guide their judgement, and may save their life. There are two aspects of this capacity to integrate information from different senses that are likely themselves synergistic. First, multisensory information may accelerate perceptual decision-making and result in faster and more accurate responses (reviewed in^1–4^). Second, multisensory information may provide a more efficient means for learning and memory than unisensory stimuli, which in turn can guide future behaviour (reviewed in^5–8^). Learning in multisensory contexts is thus of clear adaptive benefit during development and throughout the lifespan, particularly given the fact that multisensory contexts are reflective of naturalistic settings^9^. It thus logically follows that the gain afforded by multisensory processes may themselves provide a scaffold for improved higher-level cognitive functions such as learning, recognition memory, working memory, and fluid intelligence (among others) (reviewed in^10^). The aim of the present study was to assess the presence of such relationships in school-aged children.

Considerable research points to a general link between processing speed and measures of intelligence^11–14^, in adults as well as in school-aged children^15^. One potential consideration with that research is that the tasks used to evaluate processing speed were all visual in nature and thus did not assess the contribution of other sensory systems to cognitive abilities, including intelligence. At the same time, it stands to reason that individuals capable of capitalising on situations that improve processing speed (e.g. multisensory contexts) should also demonstrate stronger cognitive abilities; what Rose and colleagues refer to as a “cognitive cascade”^2,16^.

Longitudinal studies have linked cross-modal pattern matching in infants with their later reading abilities, such as in the seminal work of Birch and Belmont^17–19^ in 220 elementary (5–12 years old) scholars. This link was then extended to infants, including those born prematurely, by Rose and colleagues. An infant’s ability to match information (typically temporal patterns) between the senses is predictive of later reading skills. In particular, matching abilities *between* the senses has been shown to be a better predictor of reading skills than matching skills for patterns *within* a given sensory modality^20^. The capacity to establish sensory-independent or multisensory representations may be a core underlying skill for cognitive functions to develop and thus are indicative of core intelligence.

While the literature in infants and young children appears to support links between multisensory processes and higher-level cognition, establishing these links in school-aged children has proven more elusive. There is evidence that school-aged children (8–12 years-old) do benefit from multisensory compared to unisensory learning contexts, with facilitated later (unisensory) recognition memory^21,22^. Similar conclusions are garnered from the works of Broadbent and colleagues. These authors found incidental learning to be improved by multisensory cues^23^, and that retention of category learning over a 24‐hour delay to be significantly higher for multisensory cues than unisensory ones in 5–10 year-old schoolchildren^24^. This is consistent with literature in adults reporting evidence for links between processes subserving multisensory integration on the one hand and cognitive functions, including recognition memory, on the other hand. For example, Thelen *et al*.^25^ showed that individual performance on a continuous object recognition task could be predicted by brain responses to multisensory, but not unisensory, stimuli at initial encounters. Likewise, healthy elderly and those with mild cognitive impairment can be classified based on performance on a simple multisensory detection task, but not from unisensory performance alone, highlighting functional links between multisensory processes and memory (dys)functions^26^.

While there is evidence that children (and, later, adults) indeed garner benefits from multisensory contexts when performing memory tasks, the links between benefits of multisensory information during stimulus processing and measures of intelligence remain to be firmly established. For example, one study of 95 school children aged 6–11 years old^27^ compared performance on an auditory-visual simple detection task with scores from Raven’s Coloured Progressive Matrices^28^ and the Neal Analysis of Reading Ability^29^. In this work, there was no evidence of a statistically reliable link between multisensory facilitation of behaviour and these measures of cognitive function. Instead, those results provide evidence that multisensory processes, at least those indexed by violations of Miller’s race model inequality, remain immature in this age group. In a later study of 88 school children, Barutchu and colleagues observed a significant difference in full-scale IQ between those children whose facilitation of reaction times exceeded probability summation and those children whose multisensory facilitation could be explained by probability summation^30^. An additional more recent study of 38 8–11 years-old children reported no correlation between (absolute) multisensory reaction time facilitation and IQ scores. Instead, there was a significant positive correlation between raw multisensory reaction time and their working memory index^31^. It should be noted, though, that there was no evidence for a systematic correlation between measures of multisensory facilitation and IQ scores (In fact, there were positive correlations between IQ and unisensory RTs^27,32^; a pattern somewhat at odds with the notion of IQ being coupled with processing speed or with facilitation under multisensory conditions).

That multisensory processing capabilities are related in some manner or another to cognitive ones is certain, as is the evidence that this relationship develops (and perhaps modulates in its nature) over childhood and adolescence. As this relationship could potentially offer a long-term scaffold to improve a child’s scholastic outcomes, both in the case of typical development as well as in cases of neurodevelopmental disorders^15,33,34^, clarification seems important. Our prior work in adults would indeed suggest that the manner in which an individual detects multisensory stimuli in their environment is predictive of how well multisensory contexts will be beneficial for recognition memory functions^25,35–41^. One implication is that low-level multisensory processes may be predictive of higher-level cognitive functions, be it multisensory or more traditional and unisensory, and that such relationships may be formed during childhood (and perhaps earlier). To better understand the nature of these interactions, here, we collected data from both a simple detection task and a continuous recognition memory task, which we have used extensively in our research in adults^25,35–41^, together with standardised neuropsychological measures of working memory and fluid intelligence in school-age children.

## Materials and Methods

### Participants

In total, seventy-seven children (36 girls) from 4.6 to 15.5 years old (M_age_ = 8.1 years, SD = 3.0 years) partook in the experiment. All children had normal or corrected vision and reported no hearing loss. Moreover, Swiss children are all screened at age of 4 for sensory and learning disabilities. Any child with a reported suspicion of such disabilities was excluded from participating in our study. These individuals are the subset of participants from another study comparing pedagogical settings, and so information about schooling was also collected (Montessori and traditional). Nine schoolchildren were excluded from the study due to poor performance on the detection task (N = 3), defined as an accuracy rate lower than 30%, or due to missing data from technical issues (N = 6). The final sample included 68 children (32 girls), aged 4.6–15.5 years (M_age_ = 7.9 years, Median = 6.4 years, SD = 3.0 years). The study was conducted in accordance with the Declaration of Helsinki, and all parents provided written informed consent for their child to participate. The experimental procedures were approved by the Vaudois Cantonal Ethics Committee (Commission cantonale d'éthique de la recherche sur l'être humain).

### Tasks and procedure

All experiments took place within Swiss French-speaking schools, and a separate room was set up for testing of individual children. Two different examiners collected the data, and task order was randomized. For computerized tasks, children were seated in front of a 20”-screen laptop. The auditory stimuli were presented over headphones (model: CASIO LK-260), and the volume was adjusted to a comfortable level (~60 dB, as measured with the Decibel meter from the laptop)^42^. Both tasks were presented and controlled electronically using the E-Prime 2.0 Professional software (Psychology Software Tools, Pittsburgh, PA), and the behavioural data were recorded through the laptop’s keyboard.

#### Simple detection task

Children were presented with either visual (V), auditory (A) or audiovisual (AV) stimuli. Each child was presented with a total of 60 trials with a pseudo-randomised presentation, and equal distribution of the V, A, and AV conditions (i.e. 20 per condition). The visual stimuli were white drawings (cloud or star) presented on a black background, and the auditory stimuli were two different tones (44100 Hz digitisation; 16 bit stereo) that differed in their spectral composition to create two “opposite” types of sounds (the first one ranged from 20 Hz to 21000 Hz and the second one - from 18700 Hz to 19600 Hz). Stimuli were intermixed within blocks to maintain a high level of attention and unpredictability (in terms of which specific sensory modality would be stimulated). The audiovisual (AV) stimuli were the simultaneous and synchronous presentation of a visual and auditory stimulus. This type of detection paradigm is highly similar to that used by Fort and colleagues^43^ in their seminal work in adults. Stimulus duration was 500 ms and was followed by a randomised inter-stimulus interval (ISI) ranging from 1500 to 1900 ms, during which time a central, white fixation cross was presented. Children were asked to press a button (the keyboard spacebar) as fast as possible when they perceived any type of stimulus. Both accuracy and reaction time were recorded.

#### Continuous recognition task

Children performed a continuous recognition task, adapted from Thelen *et al*.^25^ The task was a 2-alterative forced choice that required the discrimination of initial (i.e., ‘first’) from repeated (i.e., ‘second’) instances of line drawings of common objects presented in a series of trials within a block (i.e., an “old/new” task) by pressing one of two buttons. The visual objects were black drawings presented centrally on a white background. The sounds were also selected from Thelen *et al*. (16 bit stereo; 44100 Hz digitization; 10 ms rise/fall to avoid clicks, they differed in their spectral composition, ranging from 100 Hz to 4700 Hz, and sometimes were modulated in terms of amplitude envelopes and/or waveform types). Trials were pseudo-randomised within a block of 60 trials (30 different drawings). On each trial a single image (selected from the original study) was presented alone (V) or with a congruent (AVc) or meaningless (AVm) sound (equal distribution of the three conditions; 10 trials per condition). Images were controlled to equate spatial frequency spectra and luminance between image groups (AV vs. V), according to the original task. Stimuli were presented for 500 ms, followed by a randomised inter-stimulus interval (IS) ranging from 900 to 1500 ms, where a fixation cross was shown. The mean number of trials between the initial and the repeated presentation was 5 ± 1 pictures for both V and AV conditions. Altogether, children performed four different blocks with new drawings each time (only two presentations of each drawing over all the experiment). The second presentation being always unisensory (V). Emphasis was put on both speed and accuracy. Supplementary Figure 1 illustrates the paradigm. Stimulus timing and synchrony across sensory modalities for both the simple detection task and the continuous recognition task were tested and verified using the EEG system in our laboratory as an “oscilloscope”. Visual signals were converted to voltage with a photodiode, and auditory signals were directly taken from the output of the sound card. Simultaneous stimulus presentation has been reported to be perceived as synchronous both by adults and children (e.g.^44^).

#### Working memory

Children performed the Ascending Span task from the WISC-IV^45^ to investigate the relationship between elementary multisensory processes and more complex cognitive abilities such as working memory^46^. The child was asked to listen and memorise a string of numbers spoken out loud by the experimenter and to repeat the string in an ascending order. The assessment started with a two digits string, and if the child successfully performed two trials in a row, an additional digit was added to the string. If the child missed a trial, a digit was removed from the string. If they missed three trials in a row the evaluation stopped. A final score was computed for the ascending digit task, based on the maximal number of correctly memorized and properly re-ordered digits, with a maximum of 7. No time limit was set for the answer; only accuracy was emphasized. These scores were then age-standardised based on mean span per year of age based on ref. ^47^.

#### Fluid intelligence (g factor)

Children performed the black and white version^48^ of Raven’s Coloured Progressive Matrices^28^ to assess abstract reasoning and non-verbal intelligence. It is a multiple-choice test composed of 36 items. For each item, an incomplete matrix was presented, and the child was asked to identify the missing element to complete the matrix. Participants had 15 minutes to complete as many matrices as possible. This test was conducted collectively (per small groups of maximally 5 children). Raw scores were based on the number of correct items (max. 36). The raw scores were then age-standardised using the calibration scale based on a sample of 1064 French schoolchildren following a traditional pedagogy (ECPA Pearson)^49^.

### Analysis design

As mentioned above, participants who missed more than 30% of the trials at the Simple Detection Task (3 children; mean age = 6.53, SD = 2.15), or with missing data due to technical issues (6 children; mean age = 10.28, SD = 1.10) were excluded from the analyses. Computerized data were pre-processed using Excel; correct trials with a valid RT (*subject*’ *smean RT* ± 3*SD*) were considered in analyses. Statistical analyses were run with Jamovi open-access software (retrieved from https://www.jamovi.org) as well as SPSS version 26 (IBM Corporation). Statistical significance criterion was set at p ≤ 0.05. For all tests, the effect size is reported (either partial eta squared or Cohen’s d). The full correlation matrix of the measures used in this study are provided in Supplemental Table 1.

First, to confirm multisensory benefits on a simple detection task, a repeated-measures analysis of covariance variance (ANCOVA) on mean RTs was performed with the within-subjects factor Condition (A, V, AV), and Age as the co-variate. We also performed this ANCOVA on detection rates. We also ran a repeated-measures ANCOVA on the accuracy rate [%] with the repetition conditions only from the continuous recognition task. The within subjects factor was Condition (V−, V + c, V + m) and Age was the co-variate. While previous results in adults has repeatedly indicated that RTs are not significantly modulated across conditions in this task^50^, we nonetheless also analysed RTs from the continuous recognition task in a similar ANCOVA design as described above.

Second, in order to investigate how low-level multisensory gain (simple detection task) was related to high-level (continuous recognition task) multisensory gain as well as to both working memory and fluid intelligence scores, a *relative multisensory gain* was derived from the detection task for each subject as:

$$\Delta RT[{\rm{ \% }}]=\frac{faster\,unisensory\,Mean\,RT-multisensory\,Mean\,RT}{faster\,unisensory\,Mean\,RT}\,\times \,100$$

In addition, a *relative multisensory memory gain* was computed from the continuous recognition task for congruent AV recall condition as:

$$\Delta Accuracy[{\rm{ \% }}]=({\rm{ \% }}Accuracy\,V+c)-({\rm{ \% }}Accuracy\,V\,-\,)$$

In this study, we specifically addressed the relationship between low-level multisensory processes and higher-order cognitive abilities. First, the relative *multisensory gain* value of each subject was related to the *relative multisensory memory gain* from the continuous recognition task using a stepwise linear regression with the relative multisensory memory gain as the dependent variable and relative multisensory gain and age as the independent variables. Next, we related the relative multisensory gain and age-standardised working memory scores using a logistic regression model (given the fact that the working memory scores are discrete rather than continuous). Finally, we related the relative multisensory gain with age-standardised fluid intelligence scores using a stepwise linear regression with the fluid intelligence scores as the dependent variable and relative multisensory gain and age as the independent variables. For completion and despite our specific research questions regarding the relationship of relative multisensory gain to various global cognition measures, we also include a complete correlation table across all the measures in this study.

In addition, to control for the specificity of multisensory versus unisensory processes, we also computed the *relative unisensory gain* from the detection task, as:

$$\Delta RT[{\rm{ \% }}]=\frac{slower\,unisensory\,Mean\,RT-faster\,unisensory\,Mean\,RT}{slower\,unisensory\,Mean\,RT}\,\times \,100$$

We identified the slower and the faster sensory modality for each participant, separately. In 65 of the children, the visual modality was faster. In the remaining 3 children, the auditory modality was faster. This measure of relative unisensory gain was then related to (i) the *relative multisensory memory gain* from the continuous recognition task, (ii) age-standardised working memory scores, and age-standardised fluid intelligence scores in an analogous manner to what is described above.

## Results

### Simple detection task

The children performed the simple detection task with near-ceiling performance. Mean detection rates were 93.1%, 95.1%, and 96.5% for the visual, auditory, and multisensory conditions, respectively. These data were submitted to a one-way repeated-measures ANCOVA, with Condition as the within-subjects factor and Age as the co-variate (Greenhouse-Geisser corrected degrees of freedom are reported in cases of violation of assumptions of sphericity). There was a main effect of Condition (F_(1.813,119.639)_ = 4.769, *p* = 0.012, η_p_^2^ = 0.07) and a general increase in accuracy with age (i.e., significant covariation; F_(1,66)_=14.11, *p* < 0.001, η_p_^2^ = 0.18). However, this co-variation did not reliably differ across conditions (F_(1.813,119.639)_ = 2.22, *p* = 0.12, η_p_^2^ = 0.03). Detection rates for visual stimuli were significantly lower than those for multisensory stimuli (p_bonferroni_ = 0.005, *d* = 0.36). No other contrasts were statistically significant (p’s > 0.17). Thus, and despite RTs being overall slower for A than V conditions (see below), there was no evidence that this slowing was matched by impaired detection rates.

**Figure 1:**
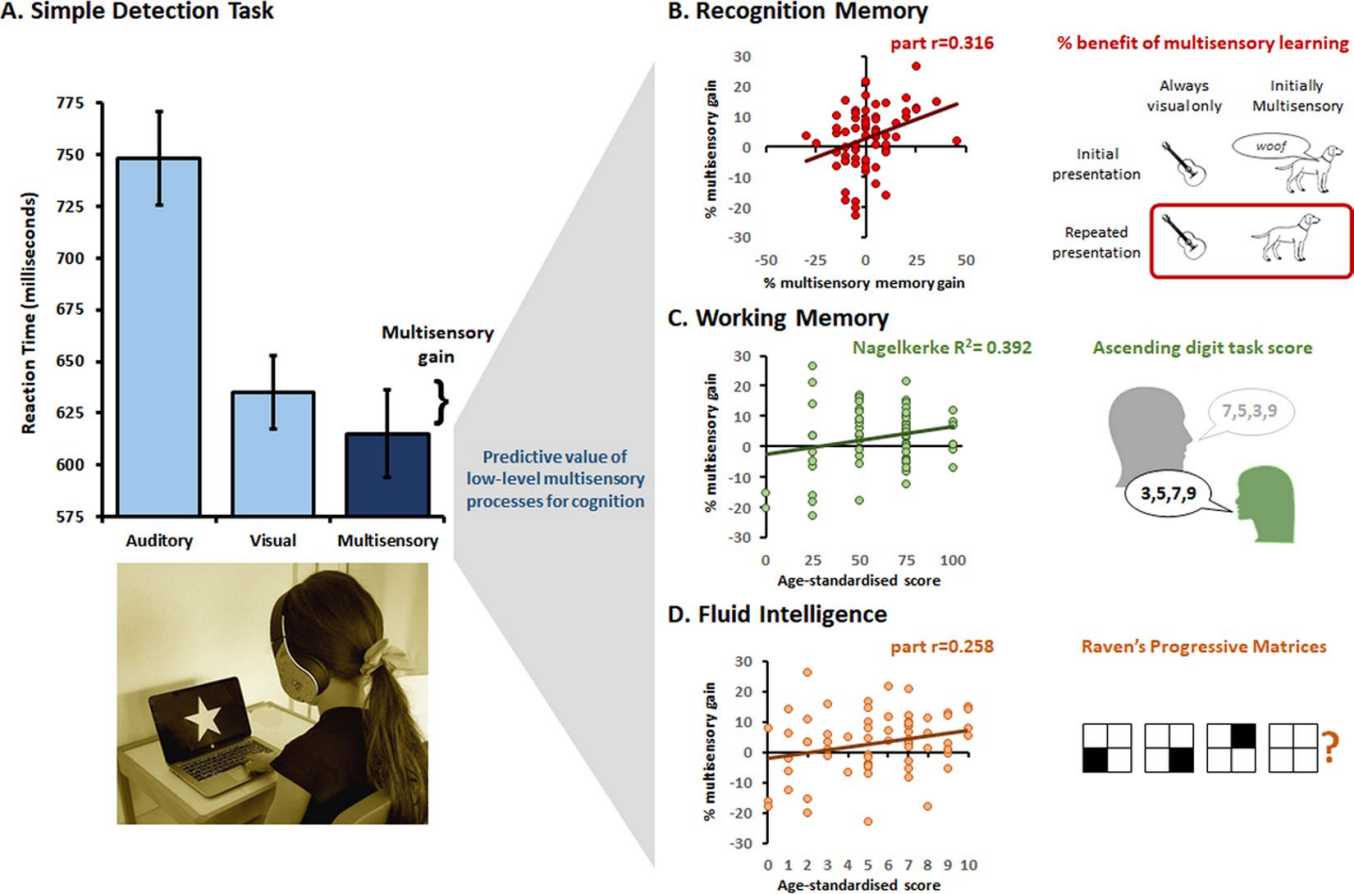
Multisensory gains in simple detection predict memory as well as fluid intelligence in schoolchildren. (**A**) Simple detection task; children were asked to press a button as fast as possible whenever a stimulus (auditory, visual or auditory-visual multisensory) was perceived. On average, reaction times were significantly faster for multisensory than for either auditory or visual stimuli (p < 0.001 and p < 0.035, respectively). For each child, a measure of multisensory gain was derived, equal to the relative difference in mean reaction time between the multisensory and the better unisensory condition. This percentage of multisensory gain (plotted on the y-axis in panels B–D) was linearly related to several measures of cognitive functioning, including recognition memory on a continuous old/new recognition task (**B**), working memory as assessed with the ascending digit task (**C**), and fluid intelligence as measured with Raven’s Progressive Matrices (**D**). The images in panel B are from the Snodgrass and Vanderwart (1980) database^85^.

Mean RTs were computed for each condition (AV, V, A) and subject (see Table 1 for group averages). Results of the one-way repeated-measures ANCOVA, with Condition as the within-subjects factor and Age as the co-variate, yielded a main effect of Condition (F_(2,132)_ = 23.53, *p* < 0.001, η_p_^2^ = 0.26) and a general decrease of RT with age (i.e., significant covariation; F_(1,66)_ = 40.50, *p* < 0.001, η_p_^2^ = 0.38). However, this co-variation did not reliably differ across conditions (F_(2,132)_ = 2.27, *p* = 0.11, η_p_^2^ = 0.00) (Fig. 1A). Post-hoc paired t-tests with a false-rate discovery (FDR) p-value correction at q = 0.05, showed participants had faster RTs on trials with AV stimuli than those with A stimuli (t_(67)_ = 12.95, *p*_*FDR*_ = 0.002, Cohen’s d = 1.57) as well as those with V stimuli (t_(67)_ = 2.14, *p*_*FDR*_ = 0.036; Cohen’s d = 0.26), and faster RTs for V than A condition (t_(67)_ = 11.58, *p*_*FDR*_ = 0.002, Cohen’s d = 1.40).

**Table 1:** Tasks’ mean scores and standard deviations.

Tasks		Mean	SD
Detection RT [ms]	*A*	748	187
	*V*	635	146
	*AV*	615	173
Multisensory gain [%]		3.19	10.17
Unisensory gain [%]		14.98	6.98
Continuous recognition Accuracy [%]	*V*−	68.8	19.5
	*V*+*c*	70.2	18.2
	*V* + *m*	69.4	17.3
Relative multisensory memory gain [%]		1.40	13.0
Age-standardised Working Memory [%]		61	25
Age-standardised Fluid Intelligence [scale]		5.42	2.81

Across participants, the average relative *multisensory gain* was 3.19%, SD = 10.2%. The average absolute multisensory gain in milliseconds was 17.78 ms, SD = 75.95 ms. These metrics were highly positively correlated, even when controlling for age (partial r_(65)_ = 0.975; p < 0.001). Across participants, the average relative unisensory gain was 14.98%, SD = 6.98%. The relative multisensory and unisensory gains (in percentages) were negatively correlated, when controlling for age (partial r_(65)_ = −0.343; p = 0.004).

It is important to mention that our paradigm, which entailed 2 visual stimuli, 2 auditory stimuli and their 4 multisensory combinations. It could be argued that one of the visual stimuli or auditory stimuli was more challenging to process, despite the task requirement of simple detection and the high performance rates of the participants. To assess this possibility, we compared mean RTs for the 2 visual stimuli, and there was no significant difference (635 vs. 626 ms; p = 0.43). We also compared mean RTs for the 2 auditory stimuli, and there was no significant difference (735 vs. 753 ms; p = 0.20). It could also be argued that participants established an implicit association between a given visual and auditory stimulus; a notion referred to as crossmodal correspondence^51^. While the fact that all multisensory combinations were equally probable provides one level of argument against this possibility, we also assessed this empirically by comparing mean RTs from what could arguably be labelled as the congruent and incongruent combinations^51^. There was no significant difference (548 vs. 562 ms; p = 0.24).

### Continuous recognition task

Accuracy rates [%] were computed for each repetition condition per subject; initially visual [V−] (mean = 68.8%, SD = 19.5%), initially paired with a meaningless sound [V + m] (mean = 69.4%, SD = 17.3%), and initially paired with a semantically congruent sound [V + c] (mean = 70.2%, SD = 17.3%). A repeated-measures ANCOVA, with Condition as the within-subjects factor and Age as the co-variate, yielded a significant covariation between accuracy and age (F_(1,132)_ = 23.0, *p* < 0.001, η_p_^2^ = 0.26). Neither the main effect of Condition (F_(2,132)_ = 1.39, *p* = 0.25, η_p_^2^ = 0.20), nor the interaction term of age co-varying differently across Condition (F_(2,132)_ = 2.03, *p* = 0.14, η_p_^2^ = 0.03) were reliable. Across participants, the average *relative multisensory memory gain* was 1.40%, SD = 13.0%, range −30% to 45%. The ANCOVA using RTs as the dependent measure did not yield a reliable main effect of Condition (F_(2,132)_ < 1) or any reliable covariation with age (F_(2,132)_ < 1).

### Predictive value of gains in simple detection for memory and global cognitive functions

We first conducted a stepwise linear regression, using the relative multisensory memory gain as the dependent, outcome variable and relative multisensory gain on the detection task as well as age as independent variables. The regression model was statistically significant (R = 0.316; F_(1,66)_ = 7.296, p = 0.009). Only the relative multisensory gain on the detection task was identified as a significant predictor of relative multisensory memory gain, accounting for 10% of the unique variance (part r = 0.316). Age did not significantly increase the performance of the model, p = 0.456). Figure 1B shows a scatterplot relating the relative multisensory gain on the detection task with that on the continuous recognition memory task.

Next, we conducted a multinomial logistic regression, using the age-standardised working memory scores as the dependent, outcome variable and relative multisensory gain on the detection task as well as age as covariates. Addition of the relative multisensory gain on the detection task and age to a model that contained only the intercept significantly improved the fit between the model and data, χ^2^(8, N = 68) = 30.90, Nagelkerke R^2^ = 0.392, p < 0.001. Significant unique contributions were made by both the relative multisensory gain on the detection task [χ^2^(4, N = 68) = 11.381; p = 0.023] and age [χ^2^(4, N = 68) = 19.724; p = 0.001]. Goodness of fit was explored by using the Pearson chi-square statistic, which was not statistically significant (p = 0.98). Figure 1C shows a scatterplot relating the relative multisensory gain on the detection task with age-standardised working memory scores.

Finally, we conducted a stepwise linear regression, using the age-standardised fluid intelligence scores as the dependent, outcome variable and relative multisensory gain on the detection task as well as age as independent variables. This regression model was statistically significant (R = 0.258; F_(1,66)_ = 4.718, p = 0.033). Only the relative multisensory gain on the detection task was identified as a significant predictor of the age-standardised fluid intelligence scores, accounting for 6.7% of the unique variance (part r = 0.258). Age did not significantly increase the performance of the model, p = 0.392). Figure 1D shows a scatterplot relating the relative multisensory gain on the detection task with age-standardised fluid intelligence scores scores.

To assess the specificity of the relative multisensory gain on the detection task as a predictor of global cognitive functions, we performed the abovementioned regressions with the relative unisensory gain on the detection task. In the case of relative multisensory memory gain, the model including age and unisensory gain as predictors did not result in a significant improvement (R = 0.175; F_(2,65)_ = 1.31; p = 0.362). In the case of age-standardised working memory scores, addition of the unisensory gain and age to a model that contained only the intercept significantly improved the fit between the model and data, χ^2^(8, N = 68) = 20.537, Nagelkerke R^2^ = 0.280, p = 0.008. Significant unique contributions were made only by age [χ^2^(4, N = 68) = 18.966; p = 0.001], but not by the unisensory gain [χ^2^(4, N = 68) = 1.016; p = 0.907]. Goodness of fit was explored by using the Pearson chi-square statistic, which was not significant (p = 0.94). In the case of age-standardised fluid intelligence scores, the model including age and unisensory gain as predictors did not result in a significant improvement (R = 0.221; F_(2,65)_ = 1.67; p = 0.196).

## Discussion

In this study, we investigated the relationship between multisensory gain in a simple detection task and global cognitive measures such as memory, working memory and fluid intelligence. Our principal finding is the statistically significant and selective link between low-level multisensory processes and multiple measures of higher-order cognitive performance in schoolchildren. These links were observed not only with laboratory-based tasks, for which the contribution of age was controlled, but also with age-standardized clinical evaluation tools that index working memory and fluid intelligence. Such links did not generalize to unisensory processes, suggestive of a certain degree of specificity of the studied constructs. These collective findings reinforce the hypothesis that multisensory perceptual processes provide a crucial scaffolding for cognition throughout the lifespan^1^.

A long history of research has reported links between unisensory as well as multisensory stimulus processing and measures of cognition and intelligence in infants and pre-school children^5,10,16,17^. However, the majority of these studies involved tasks that required matching information (e.g. shape or temporal pattern) across the senses rather than simple detection of the stimuli^19,20^, obfuscating the ability to claim that it is specifically the low-level stimulus processing mediating such links, rather than a common higher-level cognitive function contributing to both tasks. In fact, we do know that children and adults allocate attention differently to unisensory and multisensory stimuli^52,53^. In a series of studies in schoolchildren, Barutchu and colleagues did not observe a linear correlation between indices of low-level multisensory processing and intelligence scores. Specifically, in their 2009 study, they found no evidence for associations between simple reaction times to multisensory stimuli (or absolute measures of multisensory facilitation as derived from mean RTs between multisensory condition and the better unisensory condition) and non-verbal IQ (as measured with Raven’s progressive matrices) or reading abilities (as measured with the Neal Analysis of Reading Ability). Likewise, no correlation was observed in a subsequent 2011 paper that used the WISC-IV as a measure of IQ. The 2009 study focused on age-related differences in the extent to which simple reaction times were facilitated under multisensory conditions beyond what could be explained by probability summation, using Miller’s race model inequality^54^. The 2011 study revealed IQ differences between sub-groups of children according to whether or not the child’s multisensory facilitation of RTs exceeded predictions of probability summation. The present results and those of Barutchu *et al*. are consistent to the extent that they both indicate that the degree to which a child benefits from multisensory stimuli is related to their global cognition. Here, in our view, age-related differences in violations of Miller’s race model inequality, even if repeatedly reported, ought to be considered with some caution. For one, there are examples in adults where such violation have not been systematically observed^55^, raising the possibility that this metric is not fully indicative of the maturity of multisensory processes. Second, biases in the use of Miller’s inequality can be observed when numbers of trials are low^56,57^, which is often the case in studies of children. Third, non-linear neural response interactions can be decoupled from violations in Miller’s inequality^58,59^. Additional research is clearly required to fully ascertain which multisensory or integrative processes are indexed by violations of Miller’s inequality. Such notwithstanding, evidence from multiple laboratories would indeed indicate that some forms of multisensory integration remain immature in children as old as 10–11 years old. Our findings thus provide an important extension beyond what has been previously reported by Barutchu and colleagues. By showing a correlation between the percentage of multisensory facilitation of simple reaction-time and age-standardised measures of IQ, we provide evidence that multisensory processes are sufficiently mature already in school-aged children to be informative of their global cognitive abilities. Our findings also extend the studies showing that cross-modal matching is linked with cognitive functions^19,20^. We show that multisensory gains during simple detection, which arguably rely on more rudimentary processes than those involved in temporal matching, can reliably predict several measures of global cognitive functions.

It is also worth mentioning that our data are consistent with a rich literature characterising links between unisensory processing speed (as measured on either simple or choice reaction time tasks) and intelligence measures (e.g.^11,60^; reviewed in^61^). This can be gleaned from the correlation matrix (Supplementary Table 1), which generally shows a negative correlation between unisensory reaction times and working memory as well as fluid intelligence scores, though not with performance on the continuous recognition task. However, multiple regression analyses that included both RTs and age did not lead to a consistent pattern of results. While unisensory RTs reliably predicted age-standardised fluid intelligence scores, they did not predict either age-standardised working memory scores or relative multisensory memory gains on the continuous recognition task (Supplementary Table 2). Such notwithstanding, the claim in these prior studies is that simple reaction times, in general, reflect processing speed of basic cognitive operations. One prominent hypothesis focuses on the notion of neural efficiency (reviewed in^62^). More efficient processing, as in the case of individuals with higher intelligence or cognitive abilities, is paralleled by faster reaction times. It has also be shown that multisensory conditions result in less variable reaction times^63^, which may be a further contributing factor as to why multisensory processes may be particularly informative of cognitive functions. Here, our findings only reinforce the idea that low-level stimulus processing – and specifically the ability to garner benefits from multisensory contexts during low-level stimulus processing – are tightly related to higher-level cognitive processes (i.e., memory) and intellectual abilities in schoolchildren. Nonetheless, additional research will be required to not only determine to what extent multisensory processes are innate and/or experience-dependent (see^10^ for discussion), but also to what extent genetic factors contribute to multisensory stimulus processing, particularly given some evidence for genetic contributions to speed of information processing and its link to IQ (e.g.^60^). Regarding the former aspect, an ongoing clinical trial by our group is investigating multisensory processes in prematurely born infants and children as well as their predictive value for cognitive functions and scholastic achievement^64^. It is likewise important to consider the extent to which our findings are indicative of links between multisensory processes and a common (and perhaps general) cognitive construct or multiple such constructs. One access point to this issue is the pattern of relationships across the continuous recognition task, working memory task, and fluid intelligence. There was no reliable association between the relative multisensory memory gain from the continuous recognition task and age-standardised working memory scores (η = 0.291; p = 0.225) nor a correlation between such and age-standardised fluid intelligence scores (r_(66)_ = −0.027; p = 0.828). By contrast, and unsurprisingly (see^65^), age-standardised working memory and fluid intelligence measures were reliably associated (η = 0.482; p < 0.001) (see Supplementary Table 1). This overall pattern would suggest that the measures of working memory and fluid intelligence may be indexing a common cognitive construct. By contrast, the continuous recognition task is likely gauging a distinct construct. As such, it would thus appear that a child’s ability to garner multisensory benefits on a simple detection task provides an indicator of the integrity of at least two distinct cognitive systems.

There is a particularly straightforward and exciting implication of our findings; it suggests that multisensory learning, which is arguably more reflective of the sensory environment a child confronts and acts upon from birth onwards, could potentially empower cognitive development^66^. Linked to the above idea is the question of the extent to which multisensory processing abilities can be trained. This is a burgeoning field of empiric research. On the one hand, there are data showing that the so-called temporal binding window over which multisensory signals are perceptually bound is flexible and subject to learning^67,68^. This is important as the temporal binding window has been reported to be altered in a number of neurodevelopmental disorders^69^, as well as in aging^70^, and also to scale across tasks from simple detection to speech processing^71^. On the other hand, there are data showing that multisensory contexts are particularly effective for recognition memory not only in adults (reviewed in^41^), but also in schoolchildren^21–24^. That is, if multisensory processes can be trained^67^, preschool and school years may be ideal to facilitate early learning and basic perceptual skills through multisensory programs (discussed in^72^). This was already acknowledged by some educational approaches, such as Montessori Education where children work mainly through the manipulation of sensory materials^73^. Interestingly, the few quantitative existing studies in this area do report scholastic advantages for schoolchildren following a Montessori versus traditional system^74–76^. An additional, exploratory analysis of the current dataset was thus run on the children according to their schooling background and their multisensory gain on the detection task. The results revealed that Montessori scholars (half of the participants) were more likely to exhibit a multisensory gain than their peers in traditional schooling (χ^2^(1) = 5.7, *p* = *0.02*) (Fig. S2). These preliminary results call for further investigation of the topic of pedagogical tools, but already support increased efforts in multisensory enrichment targeting learning and memory processes. More generally, our results are in line with the cognitive cascade model as proposed by Rose *et al*.^16^ that focuses on the relationship between low-level multisensory processes and higher-order cognitive skills. While our sample size was modest, there was the added value of readily controlling for many demographic factors in a setting such as Switzerland. However, replication and multi-cultural studies will be required to establish the potential utility of multisensory tasks as a screening tool and multisensory enrichment as a learning aide.

It is important to mention some limitations of the present study. First, our study included a wider range of ages than other comparable studies^27,30,31^. The fact that we included younger children may be one contributing factor to the smaller average relative multisensory gain on the detection task that we observed here versus that observed in works by Barutchu and colleagues. When we considered an age range restricted to the 6–11 year-old range in Barutchu *et al*.^27^, the average relative multisensory gain on the detection task was 5%. That said, it is perhaps important to note that relative multisensory gains in studies of adults exhibit considerable inter-individual as well as between-study variability^55,77–80^. More importantly, our results provide no evidence that age was significantly contributing to any of the models using relative multisensory gain on the detection task as a predictor. Second, our study made no effort to calibrate the stimuli used in the simple detection task; RTs to auditory stimuli were slower than those to visual stimuli. Other similar work in children has used stimuli that resulted in equivalent mean RTs to both unisensory conditions^27,81^. Larger multisensory gains are obtained when the distributions to the unisensory conditions are closer to each other^63,82^. Interestingly and consistently with our prior work in adults^26^, we observed a strong negative correlation between relative multisensory gains and relative unisensory gains. While there was no evidence here that relative unisensory gains were reliable predictors of cognitive abilities, it may be that a combined metric of relative multisensory and unisensory processes may prove particularly effective should a multisensory detection task be used as a screening tool for neurodevelopmental disorders (cf.^26^ for a similar tactic in the case of mild cognitive impairment). Third, our study used a detection task that was somewhat different from that used in prior works. While prior studies used a single visual stimulus, a single auditory stimulus and their multisensory combination, the present study used 2 visual stimuli, 2 auditory stimuli, and all 4 multisensory combinations thereof. Prior studies in adults have used a detection task with multiple stimuli and did not observe differences between specific items^43^. Similarly, we found no such differences here nor any evidence for implicit crossmodal correspondences (at least with the stimulus set we used). Nonetheless, it would be informative for future research to determine what might constitute an optimal detection task design both in terms of predictive value for cognitive (dys)function and in terms of ease-of-use in schoolchildren, but also in preschoolers and infants.

The present findings of reliable links between multisensory processes and higher-level cognition cannot directly speak to their causality. Nonetheless, our results would indeed suggest that low-level multisensory processes may constitute an effective access point for the assessment of children and their cognitive development. They reinforce the possible applicability of multisensory processes to public health screening in schoolchildren. In fact, our group has already demonstrated such in the case of screening for mild cognitive impairment in the elderly based on a similar multisensory simple detection task^26^. In that study, a combined measure of sensory dominance and multisensory gain on performance reliably classified healthy elderly from those with mild cognitive impairment at level comparable with a standard clinical tool (i.e. the Hopkins Verbal Learning Task). It would be particularly promising to apply the present results in screening of (pre)school children, particularly given that multisensory processing has been shown to be selectively impaired in dyslexia (e.g.^33,83^) as well as autism (e.g.^84^, reviewed in^10^). Moreover, the detection task *per* se circumvents some of the major limitations of current screening batteries (e.g. parental report, socio-economic bias, requirement of literacy/numeracy skills). Combined with a prompt administration time, a simple detection task makes an attractive potential screening tool for pre-schoolers or pre-linguistic children.

## Supplementary information

Supplementary information
