# Supplementary material for: Multisensory Gains in Simple Detection Predict Global Cognition in Schoolchildren
Source: Sci Rep. 2020 Feb 4;10:1394. doi: 10.1038/s41598-020-58329-4 (PMC7000735; doi:10.1038/s41598-020-58329-4)
Supplement: Supplementary file 1 — Supplementary information [file 41598_2020_58329_MOESM1_ESM.pdf]

Multisensory gains in simple detection predict global cognition in schoolchildren

Solange Denervaud, Edouard Gentaz, Pawel J. Matusz*, Micah M. Murray*

	1. Age	2. Det-V	3. Det-A	4. Det-AV	5. Abs_AV	6. %_AV	7. %_uni	8. %Mem	9. WM	10. FI
1. Age(years)										
2. Detection task: Visual RT (ms)	-0.612**									
3. Detection task: Auditory RT (ms)	-0.590**	0.911**								
4. Detection task: Multisensory RT (ms)	-0.587**	0.897**	0.892**							
5. Detection task: Absolute Multisensory gain (ms)	0.186	-0.173	-0.302*	-0.573**						
6. Detection task: Relative multisensory gain (%)	0.254*	-0.163	-0.287*	-0.565**	0.938**					
7. Detection task: Relative unisensory gain (%)	-0.125	0.019	0.404**	0.181	-0.331**	-0.361**				
8. Continuous recognition task: Multisensory memory gain (%)	0.165	-0.013	-0.085	-0.159	0.353**	0.316**	-0.080			
9. Age-standardised working memory score	0.357**	-0.504**	-0.476**	-0.519**	0.270*	0.220	-0.035	-0.002		
10. Age-standardised fluid intelligence score	0.165	-0.278*	-0.192	-0.325**	0.189	0.258*	0.125	-0.027	0.422**	

Supplementary Table 1. Correlation matrix reporting Pearson's correlation coefficients between pairs of measures. * indicates p<0.05; ** indicated p<0.01

	**Predictors**		
	Visual RTs + age	Auditory RTs + age	Multisensory RTs + age
**Dependent variable**			
Continuous recognition task	Neither variable improved the model. ($F_{(2,65)}$=1.34; p=0.27)	Neither variable improved the model. ($F_{(2,65)}$=0.918; p=0.404)	Neither variable improved the model. ($F_{(2,65)}$=1.116; p=0.334)
Age-standardised working memory score	Significantly improved the fit between the model and data, χ^2(8, N=68) = 27.22, Nagelkerke R^2= 0.354, p< 0.001). However, there were no significant unique contributions by either visual RTs [χ^2(4, N=68) = 7.702; p=0.103] or age [χ^2(4, N=68) = 7.841; p=0.098]. Goodness of fit was explored by using the Pearson chi-square statistic, which was not significant (p=0.978).	Significantly improved the fit between the model and data, χ^2(8, N=68) = 24.96, Nagelkerke R^2= 0.330, p= 0.002). However, there were no significant unique contributions by either auditory RTs [χ^2(4, N=68) = 5.434; p=0.246] or age [χ^2(4, N=68) = 8.091; p=0.088]. Goodness of fit was explored by using the Pearson chi-square statistic, which was not significant (p=0.995).	Significantly improved the fit between the model and data, χ^2(8, N=68) = 26.947, Nagelkerke R^2= 0.351, p< 0.001). However, there were no significant unique contributions by either multisensory RTs [χ^2(4, N=68) = 7.425; p=0.115] or age [χ^2(4, N= 68) = 6.200; p=0.185]. Goodness of fit was explored by using the Pearson chi-square statistic, which was not significant (p=0.985).
Age-standardised fluid intelligence score	($F_{(1,66)}$=5.54; p=0.022), though age did not significantly contribute to the model (p=0.953)	Neither varibale improved the model. ($F_{(2,65)}$=1.383; p=0.258)	($F_{(1,66)}$=7.81; p=0.007), though age did not significantly contribute to the model (p=0.786)

Supplementary Table 2. Results of multiple regression analyses using mean reaction times on the detection task and age as predictors of relative multisensory memory gain on the continuous recognition task, age-standardised working memory scores, or age-standardised fluid intelligence scores.

A. **B.**

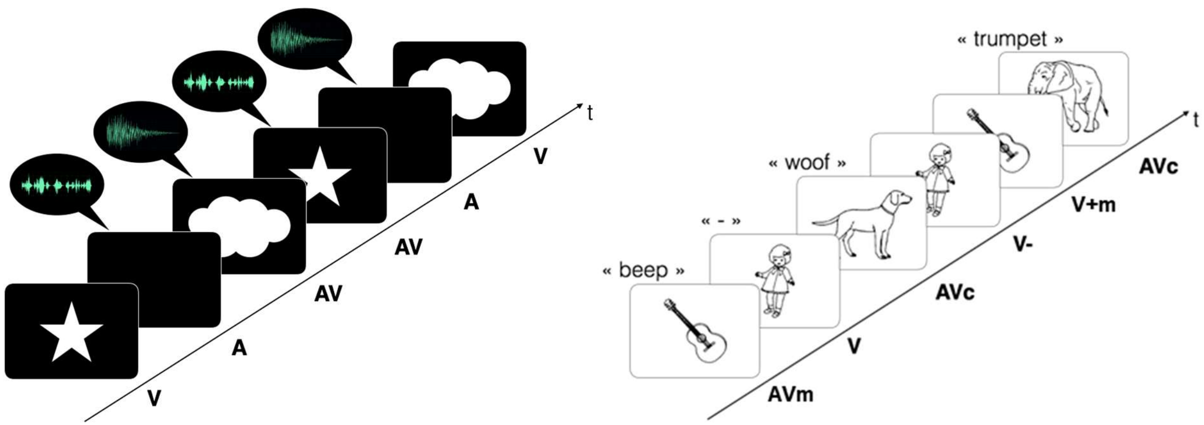

Supplementary Figure S1. Experimental design. A. Simple Detection task; V stands for visual-only sitmuli, A for auditory-only and AV for audio-visual ones. **B.** Continuous Recognition task; lines of black and white drawings taken from the Snodgrass and Vanderwart (1980) database. The first encounters were combined with meaningless (AVm), congruent (AVc) auditory sounds, or not (V). The recall conditions were always unisensory, visual only (V+m, V+c, and V-, respectively).

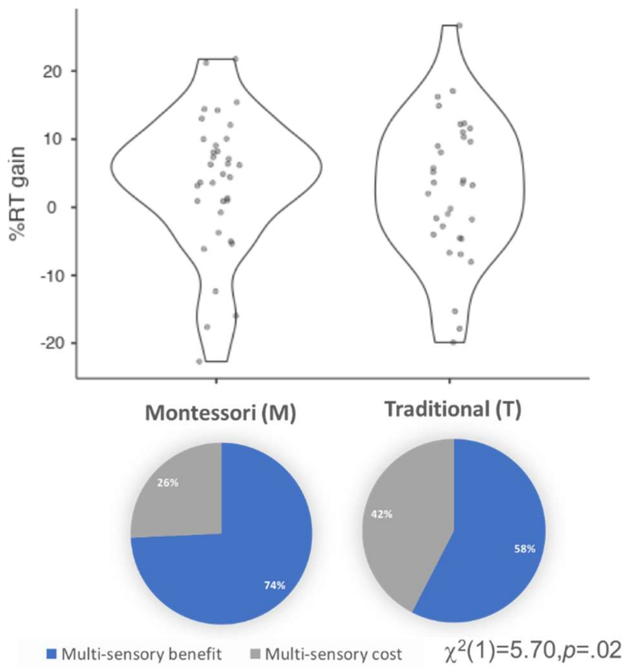

Supplementary Figure S2. The reaction time gain (*top*) according to scholastic background of the children (Montessori or traditional). By setting a threshold at zero, we could classify the scholars showing a multisensory gain (%RT>0ms) or cost (%RT<0ms). Here, the subsequent percentages per system are displayed, and statistically tested as significantly different.
